# Adherence to the Mediterranean Diet in Association with Self-Perception of Dietary Behavior (Discrepancy between Self-Perceived and Actual Diet Quality): A Cross-Sectional Study among Spanish University Students of Both Genders

**DOI:** 10.3390/nu16193364

**Published:** 2024-10-03

**Authors:** Luis M. Béjar

**Affiliations:** Department of Preventive Medicine and Public Health, School of Medicine, University of Seville, 41004 Seville, Spain; lmbprado@us.es; Tel.: +34-954-551-771

**Keywords:** Mediterranean diet, Mediterranean diet adherence, students, eating behavior, university, mobile applications

## Abstract

**Background/Objectives**: The Mediterranean diet (MD) is one of the most studied dietary patterns to date and is associated with multiple benefits for health and sustainability. However, paradoxically, adherence to the MD (AMD) has been gradually decreasing in native regions. It is necessary to identify the factors that influence AMD to reverse this trend and to mitigate the negative outcomes (for health and the environment) associated with westernized diets. The objective of this study was to assess how self-perception of dietary behavior influences AMD. **Methods**: During the 28-day repeated measurement cross-sectional study, participants’ dietary information was obtained from an initial form which established the self-perception of dietary behavior and the e12HR application to establish actual food consumption by individuals. Using the dietary information from both sources, the AMD index was calculated (specifically, the Mediterranean diet Serving Score (MDSS) index). Two categories of self-perception of dietary behavior were defined: Normal/underestimation: difference (MDSS index from initial form—MDSS index from e12HR application) ≤0; and Overestimation: difference >0 (with three subcategories: low (difference = 1–5), moderate (difference = 6–10), high (difference = 11–15)). **Results**: 139 (111, women; 28, men) Spanish university students were studied, with 98.6% (99.1%, women; 96.4%, men) falling into the overestimation category (they overestimated their dietary behavior); these students had significantly lower MDSS indexes, mean = 6.7, than students in the normal/underestimation category, mean = 12.0. Within the overestimation category, there were significant differences in the MDSS index: low (mean = 8.1), moderate (mean = 6.7), and high (mean = 4.9) subcategories and also differences that were significant in women but not in men. **Conclusions**: Overestimation of dietary behavior could be associated with lower AMD in all Spanish university students and women.

## 1. Introduction

The Mediterranean diet (MD) is a generic term used to describe the typical dietary pattern found in countries along the coast of the Mediterranean Sea; although there is no single MD, this dietary pattern presents common aspects regardless of the country in question (Crete, Greece, Italy, France, Portugal, Spain, Lebanon, Morocco, Tunisia, Turkey, and elsewhere in the Mediterranean region): It is primarily a plant-based diet (regular intake of olive oil—as the main fat—cereals, fruits, vegetables, legumes, and nuts); as for animal-based products, the intake of fish, poultry and dairy products is moderate and the intake of red or processed meat is low [1,2,3].

The MD, one of the most studied and well-known dietary patterns worldwide, has been associated with: (1) a wide range of health benefits, and (2) sustainability. Regarding the former, earlier research has provided evidence for the protective role of the MD with regards to cardiovascular disease, cancer, diabetes, excess weight/obesity, hypertension, metabolic syndrome, dyslipidemia, neurodegenerative disorders—particularly Alzheimer’s disease—respiratory diseases such as asthma and sleep apnea, renal disease, and mental disorders such as cognitive decline and depression [4,5,6,7,8]. Regarding the latter, the positive aspects of the MD on sustainability are its relatively low environmental impact in terms of water, nitrogen and carbon footprint; the richness of its biodiversity; and its positive local economic benefits [1,9,10,11,12,13].

Despite these well-known benefits (for health and the environment), paradoxically, adherence to the MD (AMD) has been gradually decreasing within its native region [12,14,15,16,17].

Given this context, it is necessary to elucidate the main factors behind the decline in AMD within its native regions to mitigate the negative outcomes (for health and the environment) associated with westernized diets. Empirical research and the theory of planned behavior suggest that behavioral attitude is an important construct associated with behavior intentions [18]. In this sense, some self-perceived attitudes of the individuals in the Mediterranean region that might influence AMD have been identified (attitudes associated with a lower AMD): attaching higher importance to the sensory appeal factor (e.g., indicating a preference for food that smells nice, has a pleasant texture, and tastes good) [19]; attaching higher importance to the convenience factor (e.g., indicating a preference for ready-to-eat or easy-to-cook products) [19]; attaching higher importance to the price factor (e.g., indicating a preference for cheaper food and good value for money) [19,20,21,22]; and a picky attitude toward eating (e.g., unwilling to eat unfamiliar foods or try novel foods) [19,23].

In a previous study carried out among cancer survivors in the United States (adults), it was found that the self-perception of dietary behavior (that is, the discrepancy between self-perceived and actual diet quality) was an important attitude that may have influenced the actual diet [18]. However, to the best of our knowledge, no previous study has explored the self-perception of dietary behavior in association with, specifically, AMD.

The primary objective of this study was to assess the influence of self-perception of dietary behavior on AMD in the whole study sample and among both genders. In the previously mentioned study conducted among cancer survivors in the United States [18], those who overestimated the self-perception of dietary behavior tended to have a lower adherence to the recommendations of the 2010 Dietary Guidelines for Americans. Following this line of thought, the research team has hypothesized that an overestimated self-perception of dietary behavior (that is, self-perceiving greater AMD than that associated with actual dietary behavior) is associated with a lower AMD among Spanish university students. Spanish university students are a particularly interesting group among the younger generations as: (1) They are a subgroup at high risk for having unhealthy dietary habits [24,25,26,27,28,29]; and (2) they constitute a very large subgroup in Spain, with 1,690,947 students currently enrolled in Spanish universities [30]. In addition, secondary objectives were to establish AMD and the prevalence of the overestimation of self-perceived dietary behavior in the whole study sample of Spanish university students among both genders.

## 2. Materials and Methods

### 2.1. Design Overview

The present study is a 28-day repeated measurement cross-sectional descriptive study.

### 2.2. Setting and Participants 

The recruitment of participants took place at the Faculties of Pharmacy and Dentistry at the University of Seville (Andalusia, Spain, South of Europe), selecting the three classrooms in which the principal investigator of the study taught (two classrooms at the Faculty of Pharmacy and one classroom at the Faculty of Dentistry); the recruitment of participants took place over two different months of the Academic Year 2023/2024: September 2023 (for the Faculty of Pharmacy) and February 2024 (for the Faculty of Dentistry).

In each of the three classrooms selected for the research, the principal investigator of the study showed the project to the students in person, explaining the objectives, risks, and benefits of the research as well as the procedures to properly fill out the different questionnaires used in the study (questionnaires in “Word” document format and questionnaires through the e12HR application (see Section 2.4. e12HR Application subsection)); finally, information was provided on how to participate in the study.

Inclusion criteria: Both genders, over 18 years old, student of Pharmacy or Dentistry (University of Seville) and possess a smartphone. 

Exclusion criteria: Food intolerances, chronic pathologies, or pregnancy (due to the possibility of requiring specialized dietary recommendations). 

Confidentiality in the research was guaranteed in accordance with the Organic Law on the Protection of Personal Data (15/1999 of 13 December, OLPPD) and the Law 14/2007 on Spanish Biomedical Research. 

The steps to participate in the study were as follows:

(1) Interested students were provided with a personal alphanumeric code in class;
(2) All documents related to the study were available on an educational platform used by the University of Seville. This educational platform is separate for each classroom (with access limited only to the students and professors of each classroom). The documents available to students on the educational platform were as follows: (a) Informed consent; (b) Initial form (see 2.3. Initial Form subsection); (c) User manual with detailed information for downloading and using e12HR, an application that is free to download from the App Store or Play Store. The documents (a) & (b) were to be filled out and submitted on the educational platform; in each of the two documents, each student had to include his or her personal alphanumeric code;
(3) The research team reviewed the documents (a) and (b) after these were returned to the educational platform. When both documents were filled out correctly, the research team activated the personal alphanumeric code so that the corresponding student had access to the e12HR application (in this way, students could not start using the e12HR application until they had completed the two documents required for the research). Once the personal alphanumeric code was activated by the research team, the student only had to open the e12HR application to start using it (see Section 2.4. e-12HR Application subsection).

To incentivize participation in the present study, taking part in the study was included as one of the class activities for the subjects “Public Health” (Faculty of Pharmacy) and “Epidemiology and Public Health” (Faculty of Dentistry) at the University of Seville; in any case, participation in the study was an optional class activity and was not evaluated for the final marks of the study subjects.

### 2.3. Initial Form

The initial form presented two sections: (1) A section with personal information which allowed the students to provide data such as their date of birth, gender, weight, height, smoking status, faculty, and type of residence; and (2) a section with dietary information which allowed the students to provide information about their self-perception of dietary behavior for certain food groups related to the MD over the previous six months. In this last section, there was information about food groups related to the MD, and consumption recommendations for each food group. The student had to indicate whether or not he or she had met the consumption recommendations for each food group over the previous six months; to do so, the student had to mark one of the two available options in the form: option 1: “Yes; I have complied with the recommendation”; or option 2: “I have not complied with the recommendation” (see Appendix A, Table A1 for the full form with dietary information).

As previously mentioned, students filled out and returned the initial form (including their personal alphanumeric code) in a “Word” document format to the educational platform. The research team converted the information from the initial form into “Excel” format for the subsequent statistical analysis. 

### 2.4. e12HR Application

e12HR is an application that allows for the long-term collection of data on the dietary intake of food groups (related to MD); this application is basically a modified 24-h recall which has been previously validated—using food frequency questionnaires (FFQ) and dietary records as reference methods—for determining the actual consumption of certain food groups related to MD [31,32].

**Figure A1:**
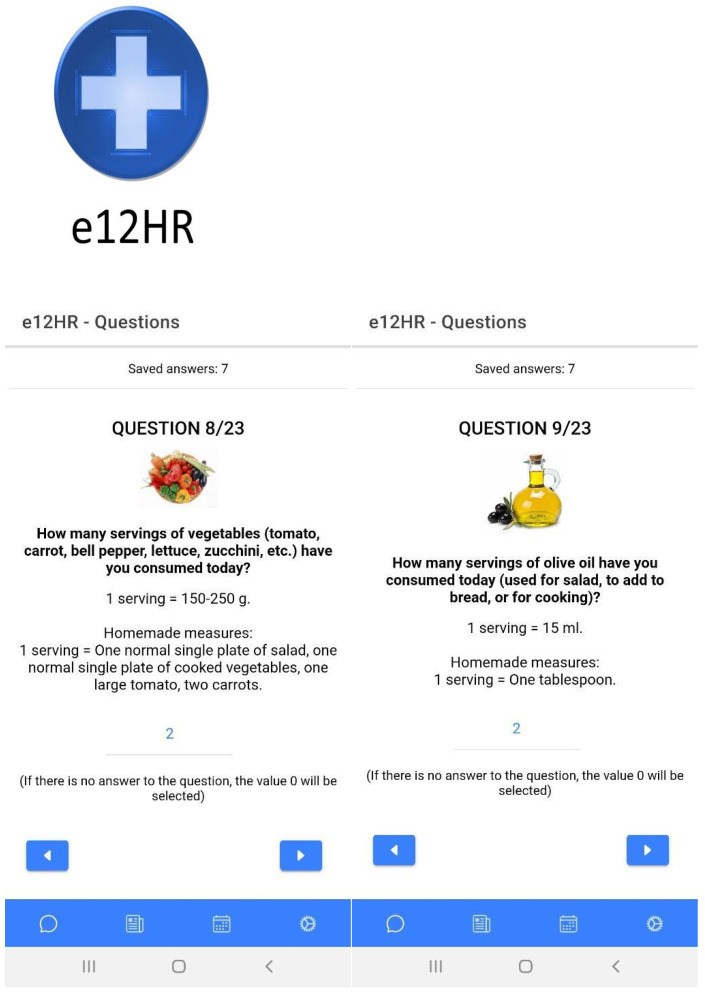
Real images of e12HR application.

The structure and functions of the version of the e12HR application used in this study have been described in detail by Béjar et al. (in “e12HR App” in Section 2.2.) [33]. In brief, this version of the e12HR application allowed participants to register the number of servings consumed during the day for each of the nineteen food groups included in the study (see Appendix A, Figure A1 for real images of the e12HR application). After completing the daily form on the application, the collected data was automatically sent to the research team’s website. The research team converted the information from the website into “Excel” format for subsequent statistical analysis.

**Figure 1:**
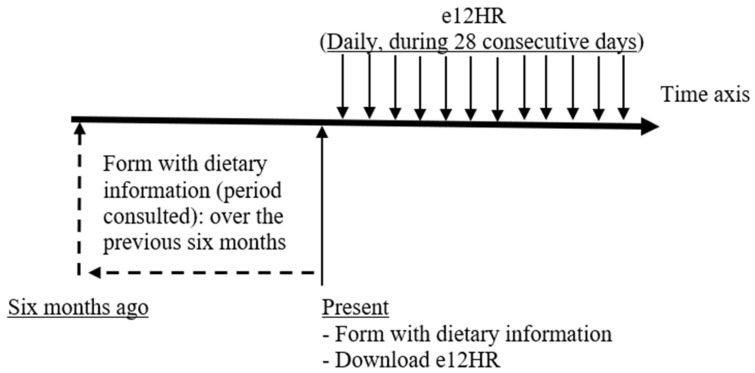
Assessment processes using the form with dietary information and e12HR application.

The study protocol in the present study (which combined two assessment processes using the form with dietary information and the e12HR application) is shown in Figure 1.

### 2.5. Adherence to Mediterranean Diet Assessment

In this study, the data from the initial form (specifically, the form with dietary information) and the e12HR application were compared. With the dietary information from both sources, AMD was calculated following the original rules of the Mediterranean diet Serving Score (MDSS) index [34]: (1) Selection of a series of key food groups (compatible with the MD); (2) establishment of dietary intake recommendations for each key food group; (3) a numerical score assigned to each item in the case of compliance with the recommendations (if the indicated recommendations were not followed, a value of zero was assigned for that item). However, it was necessary to make some modifications in order to adapt them to the characteristics of the form with dietary information and the e12HR application (Table 1).

With the dietary information from both sources (the form with dietary information and the e12HR application), scores for the MDSS index were calculated manually by the research team (using the rules shown in Table 1 and the documents in “Excel” format generated from each source). To complete the process, the value of each food group was added up and a score on the MDSS index was generated, which could vary between zero and twenty-four. In addition, the score on the MDSS index was related with one of three levels of MDSS [35]: low (0–8 points), moderate (9–15 points) or high (16–24 points).

In order to calculate the MDSS index, the daily dietary data from the e12HR application were processed as follows: (1) For each food group, the sum of the daily rations was recorded; (2) the previous sum was then divided by the number of days for which the task was completed (to obtain the average number of servings/day for each food group); and (3) for food groups which used the weekly recommendations, as opposed to daily, the result from the previous operation was multiplied by seven (to obtain the average number of servings/week).

### 2.6. Self-Perception of Dietary Behavior by Students: Overestimation or Normal/Underestimation

In this study, two categories have been defined for the self-perception of dietary behavior by students (overestimation or normal/underestimation), considering the following criteria [18]: (1) normal/underestimation category: MDSS index calculated from the data obtained from the form with dietary information—MDSS index calculated from the data obtained from the e12HR application ≤0. For the students in this category, the research team interpreted that they self-perceived equal or lower AMD than that associated with their actual dietary behavior; (2) overestimation category: MDSS index calculated from the data obtained from the form with dietary information—MDSS index calculated from the data obtained from the e12HR application >0. For the students in this category, the research team interpreted that they self-perceived greater AMD than that associated with their actual dietary behavior. For the overestimation category, three subcategories were established: low (difference from 1 to 5); moderate (difference of 6–10), and high (difference of 11–15). No differences greater than 15 were observed in the present study.

### 2.7. Statistical Analysis

Discrete variables are presented as a number followed by percentages. Continuous variables are presented using means and standard deviations (SD) and median and interquartile range (IQR).

The data were tested for normality using the nonparametric Kolmogorov–Smirnov test.

For paired samples: (1) Student’s *t*-test or the nonparametric Wilcoxon test were used for the analysis of quantitative variables; and (2) Wilcoxon test was used for the comparison of ordinal qualitative variables.

For unpaired samples: Student’s *t*-test or the nonparametric Mann–Whitney U-test was used for the analysis of quantitative variables, penalizing *p*-values with Bonferroni adjustment for multiple comparisons. 

The results were considered significant if *p*-value < 0.05, except for multiple comparisons using Bonferroni penalization: *p*-value < 0.017 (0.05/3).

Statistical analyses were performed using the SPSS statistical software package version 29.0 (SPSS Inc.; Chicago, IL, USA).

## 3. Results

### 3.1. Sample and Adherence to the Study

In the three classrooms selected for the study (two classrooms at the Faculty of Pharmacy and one classroom at the Faculty of Dentistry) there were a total of 205 students, of whom 162 signed the informed consent forms. However, two students were excluded (one due to diabetes and another due to anorexia). Of those who fulfilled the inclusion requirement for the study, 21 were considered non-responsive (as they completed the task on the app for fewer than 20 days); therefore, the study response rate was 86.9% (139/160). The data for the non-responsive individuals was not included in the later statistical analysis. No significant statistical differences were observed in the variables studied between responsive and non-responsive participants. 

### 3.2. Personal Information of the Participants

Table 2 shows the personal information of the participants. 

The 139 participants who completed the study (28-day follow-up period) registered their daily consumption for the nineteen food groups included in the study. This represents a collected total of 73,948 data points on daily consumption for the food groups (139 participants, a 28-day follow-up period, and 19 food groups included). 

### 3.3. Scores and Levels of the Mediterranean Diet Serving Score Index

Table 3 shows the scores of the MDSS index and Table 4 shows the levels of the MDSS index (in the whole study sample and by gender), calculated from the data obtained from the two sources used in the study: the form with dietary information (filled out by participants at the beginning of the study) versus the e12HR application (used by participants for the 28-day follow-up period) (see Figure 1). 

For the whole study sample and both genders, there were significant statistical differences between the indices calculated using the form with dietary information versus the e12HR application, with higher a MDSS index calculated from the data obtained from the form with dietary information. From the form with dietary information, the MDSS index was ≥14.0 (mean and median); from the e12HR application, the MDSS index was ≤7.0 (mean and median) (Table 3). 

For the whole study sample and both genders, there were significant statistical differences between the indices calculated using the form with dietary information versus the e12HR application. A higher percentage of participants with a high/moderate MDSS index was calculated using the from data obtained from the form with dietary information: From the form with dietary information, the percentage of participants with a high/moderate MDSS index was >46.0 and ≥45.0, respectively. From the e12HR application, the percentage of participants with a high MDSS index was 0.0, and the percentage of participants with a moderate MDSS index was <25.0 (Table 4).

### 3.4. Distribution of Self-Perception of Dietary Behavior by Students: Overestimation or Normal/Underestimation

Table 5 shows the distribution of the participants and the scores of the MDSS index (calculated from the data obtained from the e12HR application) by overestimation (low, moderate, and high subcategories) and normal/underestimation categories. Table 6 shows a comparison of the MDSS across the same categories/subcategories. 

For the whole study sample and both genders, most students were in the overestimation category (with percentages >96.0%), mainly in the moderate subcategory (with percentages >45.0%) (Table 5).

For the whole study sample and for women, there were significant statistical differences in the MDSS index in the normal/underestimation category (with mean values of 12.0 and 13.0) as compared to the overestimation category (with mean values of 6.7); within the overestimation category, there were significant statistical differences in the MDSS index comparing low (with mean values of 8.1 and 8.2), moderate (with mean values of 6.7) and high (with mean values of 4.5 and 4.9) subcategories. No significant statistical differences were observed for men (Table 6).

## 4. Discussion

### 4.1. Principal Findings

The main findings of the study were:

(1) The actual dietary behavior in the sample was poor: The mean score of the MDSS index was 6.8 for all students (6.8 for women and 7.0 for men) (Table 3), with 76.3% of all students at the low level of the MDSS index (75.7% for women and 78.6% for men) (Table 4);
(2) The self-perception of dietary behavior in the sample was significantly higher: the mean score of the self-perceived MDSS index was 14.3 for all students (14.3 for women and men) (Table 3), with 95.7% of all students at the moderate-high level of self-perceived MDSS index (95.5% for women and 96.4% for men) (Table 4);
(3) The vast majority of the sample overestimated dietary behavior: 98.6% of all students were in the overestimation category (99.1% for women and 96.4% for men) (Table 5);
(4) As we hypothesized, an overestimated self-perception of dietary behavior in the sample (that is, self-perceiving greater AMD than that associated with their actual dietary behavior) was associated with a lower AMD: The mean score of the MDSS index was significantly higher among students falling into the normal/underestimation category (12.0) compared to the overestimation category (6.7). Within the overestimation category, the mean score of the MDSS index grew significantly lower when moving from the low overestimation subcategory (8.1) to the moderate overestimation subcategory (6.7) and to the high overestimation subcategory (4.9). A similar trend was observed in women, with the following mean scores of the MDSS index by categories and subcategories: normal/underestimation category (13.0), overestimation category (6.7); within the overestimation category, low overestimation subcategory (8.2), moderate overestimation subcategory (6.7), and high overestimation subcategory (4.5) (Table 6). Comparisons of the normal/underestimation category versus the overestimation category must be made with caution, due to the small number of students in the normal/underestimation category (Table 5). The comparisons (between categories and subcategories) of the mean scores of the MDSS index were not statistically significant in men (Table 6); however, these comparisons must be made with caution, owing to the small number of male students (Table 2) currently enrolled at the University of Seville, Faculties of Health [30], which is actually a reflection of the small proportion of male students (28.2%).

### 4.2. Comparison with Prior Work

The results obtained in the present study were similar to the results of other studies using the e12HR application, which have been carried out by the research team over the last few years to determine the actual dietary behavior in Spanish university students (Faculties of Medicine, Pharmacy and Communication at the University of Seville): The mean scores of the MDSS index were 8.7 [33], 7.9 [36], and 7.1 [37], with 52.1% [33], 63.3% [36], and 73.8% [37] at the low level of MDSS index.

This work is pioneering since, to the best knowledge of the research team, it is the first to assess the influence of self-perception of dietary behavior on AMD in adults. There is, however, a study conducted by Xue et al. [18] that also assessed the discrepancy between self-perceived and actual diet quality (in this case, among cancer survivors in the United States), and its association with diet quality. The differences between the present study and that of Xue et al. [18] are important, nonetheless. First of all, there are the study samples: Spanish university students versus cancer survivors in the United States. Secondly, in the present study, AMD was assessed using the MDSS index (a range of scores from 0 to 24 points), while in the study of Xue et al., adherence to the recommendations of the 2010 Dietary Guidelines for Americans was assessed using the Healthy Eating (HE) index score (the HE index is a measure of dietary quality according to the federal dietary guidance of the United States, which presents a range of scores from 0 to 100 points). Despite the differences between both studies, the findings are consistent regarding the prevalence of the overestimation of self-perceived dietary behavior and its possible association with lower diet quality: (1) Both studies found a high prevalence of overestimation of self-perceived dietary behavior: 56.1% (previous study) and 98.6% (present study); and (2) the overestimation of self-perceived dietary behavior was significantly associated with lower diet quality (compared with normal/underestimation): +12.5 points in the HE index (previous study) and +7.5 points in the MDSS index (present study). These results reflect the difficulty in self-assessing diet and its possible influence on the actual diet.

### 4.3. Comparison of Dietary Information: Initial Form Versus e12HR Application

As previously mentioned, in this study the research team compared the data from the initial form (specifically, the section with dietary information) and the e12HR application; the data obtained from the form with dietary information allowed the team to establish the self-perception of dietary behavior for certain food groups related to the MD for the students, and the data obtained from the e12HR application established the actual dietary behavior of the students for the same foods groups.

The form with dietary information refers to dietary behavior over the previous six months; if a shorter period was considered in a sample of university students, the dietary pattern could be affected by specific situations, for example, school events such as exam or internship periods, or vacations. The e12HR application collects dietary behavior over the following 28 days (the time period considered in previous studies where e12HR has been used to determine the usual dietary pattern in adults [33,36,37,38]) (Figure 1). Therefore, the periods used to compare the AMD (self-perception of dietary behavior versus actual dietary behavior) were different. 

A key aspect of the research methodology followed in this study was the selection of the periods to be compared, as they had to be different but very close (preferably two consecutive periods). As such, the research team considered two scenarios, the first of which was comparing different periods. If the same period was used for the two methods, the e12HR application would be filled out first (for twenty-eight consecutive days) and the form with dietary information would be filled out at the end of this period. In this situation, it is likely that the student would be able to remember the information collected in the application during the 28-day period and that this reminiscence would make it easier to remember the dietary pattern for students (which would artificially improve their self-perception of their dietary pattern). However, the research team wanted to know the students’ self-perceptions of their dietary pattern under normal conditions. The form with dietary information is a simplified semiquantitative FFQ, based on a semiquantitative FFQ previously validated for the Spanish population [39]. It should be noted that when using FFQs, collecting data on past dietary patterns may be influenced by the most recent dietary pattern [40,41,42,43]. The second scenario was to compare very close periods, preferably two consecutive periods. In this way, as they are very close, it seemed unlikely that the dietary patterns of students would have changed significantly from one period to the next. 

### 4.4. Selected Sample: Health Sciences University Students

As previously mentioned, Spanish university students are a particularly interesting group among younger generations at high risk of having unhealthy dietary habits [24,25,26,27,28,29] and they are a very large subgroup in Spain [30]. However, all participants in this study were health sciences university students.

Health sciences university students were selected, firstly, because the students who participated in this study constitute a convenient sample (they were selected in the classes where the principal investigator of the study taught, the Faculties of Pharmacy and Dentistry), but mainly because these students constitute a special group to carry out dietary studies.

In this vein, health sciences university students have low AMD [33,36,37], and it is possible that this dietary behavior will be maintained into adulthood. While this would represent a great inconvenience for any individual in the population, it is especially so for health sciences university students [44]. The reason for this is because one of the main tasks of these students (future health sciences professionals) should be the promotion of healthy habits in the population (for example, healthy eating); and to do so, they should know which habits are considered healthy and, in addition, practice them [45]. 

The results of this study among health sciences university students (in all students and in women) suggest that it is urgently necessary to raise awareness among future health professionals of their overly optimistic attitudes in the self-perception of dietary behavior (i.e., the overestimation of self-perceived dietary behavior). This could likely improve their nutritional habits for the future, which, at the same time, could positively influence the dietary habits of their future patients. The health sciences faculty might constitute the ideal institution for implementing interventions that contribute to this end [46,47]. 

### 4.5. Limitations

This study presents several limitations: (1) The form with dietary information and the e-12HR application are self-reporting methods (respectively, FFQ and 24-h recall) and present inherent limitations amply described in the scientific literature [40,42,43,48,49,50,51]; (2) furthermore, as previously mentioned, the number of students in the normal/underestimation category (*n* = 2), as well as the number of male students (*n* = 28), were small; and (3) it was not possible to control the self-perceived attitudes of individuals that could influence AMD (which have been previously identified), such as assigning higher importance to the sensory appeal factor [19]; the convenience factor [19]; the price factor [19,20,21,22]; and a picky attitude toward eating [19,23]. This is due to the fact that the abovementioned self-perceived attitudes of the individuals were not formally collected in this study. 

### 4.6. Future Research Related to the Current Study

Future research should clarify the possible effect of the overestimation of self-perceived dietary behavior on AMD. For that, future research will focus on remedying the limitations of the present study (except for those limitations inherent to the self-reporting methods used). 

The research team intends to assess the influence of the self-perception of dietary behavior on AMD by controlling other self-perceived attitudes of individuals which could influence AMD, in health sciences university students, in both women and men, and with a larger sample size of men. Other samples of populations such as health professionals, non-health science students and the general population will also be considered. 

For the more distant future, the research team will considerer designing and evaluating specific interventional measures to raise awareness of the overestimation of self-perceived dietary behavior.

## 5. Conclusions

The Spanish health sciences university students presented low AMD (measured as the mean score of the MDSS index and the percentage of participants with a low MDSS index). The vast majority of the Spanish health sciences university students overestimated their dietary behavior. Finally, an overestimated self-perception of dietary behavior could be associated with a lower AMD (in all Spanish university students and in women). 

The results of this study suggest that nutrition interventions aimed at reducing the discrepancy between self-perception and actual diet quality have the potential to improve AMD among Spanish health sciences university students (in all students and in women).

## Figures and Tables

**Table 1:** Mediterranean diet Serving Score index for the form with dietary information and e12HR.

Food Group	Recommendation	Score (Form with Dietary Information and e-12HR)
Scoring of Food Groups Calculated on a daily basis		
Fruits	≥3 serving/day	3
Vegetables	≥3 serving/day	3
Cereals	3–6 serving/day	3
Olive oil	3–4 serving/day	3
Milk and dairy products	2–3 serving/day	2
Fermented beverages	1–2 serving/day	1
Scoring of food groups calculated on a weekly basis		
Nuts	3–7 serving/week	2
Potatoes	≤3 serving/week	1
Legumes	≥2 serving/week	1
Eggs	3–4 serving/week	1
Fish	≥2 serving/week	1
White meat	2–3 serving/week	1
Red and processed meat	≤2 serving/week	1
Sweets	≤2 serving/week	1
Total maximum score		24

Cereals: breakfast cereals, pasta, rice and bread. Olive oil: used on salads or bread or for frying. Milk and dairy products: milk, yogurt and cheese. Fermented beverages: wine and beer. White meat: poultry. Red meat: pork, beef and lamb. Sweets: sugar, candies, pastries, sweetened fruit juices and soft drinks.

**Table 2:** Characteristics of study participants.

Characteristics	n (%)	Mean (SD)	Median (IQR)
Participants who completed the study	139 (100)	- *	-
Gender		-	-
Females	111 (79.9)	-	-
Males	28 (20.1)	-	-
Age (Years)		21.3 (4.5)	19.9 (2.7)
<20	70 (50.4)	-	-
≥20	69 (49.6)	-	-
Studies		-	-
Pharmacy	89 (64.0)	-	-
Dentistry	50 (36.0)	-	-
BMI (kg/m^2^)		22.2 (3.7)	21.5 (3.7)
<25	118 (84.9)	-	-
≥25	21 (15.1)	-	-
Smoking Status		-	-
No	114 (82.0)	-	-
Yes	25 (18.0)	-	-
Physical activity status (m/w)		-	-
≥150	93 (66.9)	-	-
<150	46 (33.1)	-	-

* Not applicable. SD: standard deviation. IQR: interquartile range. BMI: body mass index. m/w: minutes/week.

**Table 3:** Scores of the Mediterranean diet Serving Score index (form with dietary information versus e12HR application).

	Form with Dietary Information		e12HR Application		
	Mean (SD)	Median (IQR)	Mean (SD)	Median (IQR)	*p*-Value *
All	14.3 (3.4)	14.0 (5.0)	6.8 (2.7)	7.0 (3.0)	<0.001
Females	14.3 (3.4)	15.0 (5.0)	6.8 (2.8)	7.0 (3.0)	<0.001
Males	14.3 (3.4)	14.0 (5.0)	7.0 (2.1)	7.0 (3.0)	<0.001

MDSS: Mediterranean diet Serving Score. SD: standard deviation. IQR: interquartile range. *p*-value: differences from the data from the form with dietary information versus the e12HR application. * Paired Student *t*-test. A *p*-value < 0.05 is considered significant.

**Table 4:** Levels of the Mediterranean diet Serving Score index (form with dietary information versus e12HR application).

	Form with Dietary Information			e12HR Application			
	n (%)			n (%)			
	High	Moderate	Low	High	Moderate	Low	*p*-Value *
All	69 (49.6)	64 (46.0)	6 (4.3)	0 (0.0)	33 (23.7)	106 (76.3)	<0.001
Females	56 (50.5)	50 (45.0)	5 (4.5)	0 (0.0)	27 (24.3)	84 (75.7)	<0.001
Males	13 (46.4)	14 (50.0)	1 (3.6)	0 (0.0)	6 (21.4)	22 (78.6)	<0.001

MDSS: Mediterranean diet Serving Score. *p*-value: differences from the data from the form with dietary information versus e12HR application. * Paired Wilcoxon test. A *p*-value < 0.05 is considered significant.

**Table 5:** Distribution of the participants and scores of the Mediterranean diet Serving Score index by overestimation (low, moderate and high subcategories) and normal/underestimation categories.

		MDSS Index (from e12HR Application)	
Category	n (%)	Mean (SD)	Median (IQR)
All			
Normal/underestimation	2 (1.4)	12.0 (1.4)	12.0 (- *)
Overestimation	137 (98.6)	6.7 (2.6)	7.0 (3.0)
Within overestimation category			
Low	41 (29.5)	8.1 (2.8)	8.0 (4.0)
Moderate	67 (48.2)	6.7 (2.2)	7.0 (3.0)
High	29 (20.9)	4.9 (2.2)	5.0 (3.0)
Gender			
Female			
Normal/underestimation	1 (0.9)	13.0 (-)	13.0 (-)
Overestimation	110 (99.1)	6.7 (2.8)	7.0 (3.0)
Within overestimation category			
Low	35 (31.5)	8.2 (2.9)	8.0 (4.0)
Moderate	51 (45.9)	6.7 (2.3)	7.0 (3.0)
High	24 (21.6)	4.5 (2.1)	4.0 (3.0)
Male			
Normal/underestimation	1 (3.6)	11.0 (-)	11.0 (-)
Overestimation	27 (96.4)	6.8 (2.0)	7.0 (3.0)
Within overestimation category			
Low	6 (21.4)	7.7 (2.2)	7.0 (4.0)
Moderate	16 (57.1)	6.6 (1.9)	7.0 (3.0)
High	5 (17.9)	6.6 (2.3)	7.0 (4.0)

* Not applicable. MDSS: Mediterranean diet Serving Score. SD: standard deviation. IQR: interquartile range.

**Table 6:** Mediterranean diet Serving Score index: comparison across overestimation (and low, moderate, and high subcategories) and normal/underestimation categories.

MDSS Index (from e12HR Application)		
Category Mean (SD)		*p*-Value
All		
Normal/underestimation	Overestimation	0.008 **
12.0 (1.4)	6.7 (2.6)	
Multiple comparisons within overestimation category		
Low overestimation 8.1 (2.8)	Moderate overestimation 6.7 (2.2)	0.004 ***
Low overestimation 8.1 (2.8)	High overestimation 4.9 (2.2)	<0.001 ***
Moderate overestimation 6.7 (2.2)	High overestimation 4.9 (2.2)	<0.001 ***
Females		
Normal/underestimation 13.0 (− *)	Overestimation 6.7 (2.8)	0.018 **
Multiple comparisons within overestimation category		
Low overestimation 8.2 (2.9)	Moderate overestimation 6.7 (2.3)	0.010 ***
Low overestimation 8.2 (2.9)	High overestimation 4.5 (2.1)	<0.001 ***
Moderate overestimation 6.7 (2.3)	High overestimation 4.5 (2.1)	<0.001 ***
Males		
Normal/underestimation 11.0 (−)	Overestimation 6.8 (2.0)	0.071 **
Multiple comparisons within overestimation category		
Low overestimation 7.7 (2.2)	Moderate overestimation 6.6 (1.9)	0.260 ***
Low overestimation 7.7 (2.2)	High overestimation 6.6 (2.3)	0.449 ***
Moderate overestimation 6.6 (1.9)	High overestimation 6.6 (2.3)	0.486 ***

* Not applicable. MDSS: Mediterranean diet Serving Score. SD: standard deviation. ** Unpaired Mann–Whitney U-test. *** Unpaired Student’s *t*-test. A *p*-value < 0.05 is considered significant, penalizing *p*-value with Bonferroni adjustment for multiple comparisons: *p*-value < 0.017 (0.05/3).

## Data Availability

The raw data supporting the conclusions of this article will be made available by the authors on request.

## Abbreviations

AMD	adherence to the Mediterranean diet
BMI	body mass index
FFQ	food frequency questionnaire
IQR	interquartile range
MD	Mediterranean diet
MDSS	Mediterranean diet serving score
OLPPD	Organic Law on the Protection of Personal Data
SD	standard deviation

## Appendix A

Please mark the suitable option for each food group by placing an “x” in the appropriate column (column “Yes; I have complied with the recommendation” or column “I have not complied with the recommendation”).

Please consider your food intake over the previous six months.

**Table A1:** Form with dietary information (by food groups related to Mediterranean Diet).

Food Group	Recommendation Yes; I Have Complied with the Recommendation	I have Not Complied with the Recommendation
	Daily consumption recommendations	
Fruits	>3 serving/day	
Vegetables	3 serving/day	
Cereals	3–6 serving/day	
Olive oil	3–4 serving/day	
Milk and dairy products	2–3 serving/day	
Fermented beverages	1–2 serving/day	
	Weekly consumption recommendations	
Nuts	3–7 serving/week	
Potatoes	3 serving/week	
Legumes	2 serving/week	
Eggs	3–4 serving/week	
Fish	2 serving/week	
White meat	2–3 serving/week	
Red and processed meat	2 serving/week	
Sweets	2 serving/week	

Cereals: breakfast cereals, pasta, rice and bread. Olive oil: used on salads or bread or for frying. Milk and dairy products: milk, yogurt and cheese. Fermented beverages: wine and beer. White meat: poultry. Red meat: pork, beef and lamb. Sweets: sugar, candies, pastries, sweetened fruit juices and soft drinks.

In order to assist in estimating the number of standard servings consumed, each food group was accompanied by an explanatory text with different homemade measurements (as the research team considered that it would be more appropriate and easier to follow for people without experience in dietetics).

The standard servings used in this form are based on a semiquantitative food frequency questionnaire previously validated for the Spanish population [39].

The standard servings used by e12HR are based on a semiquantitative food frequency questionnaire previously validated for the Spanish population [39].
